# CD8^+^ T cell cytotoxicity mediates pathology in the skin by inflammasome activation and IL-1β production

**DOI:** 10.1371/journal.ppat.1006196

**Published:** 2017-02-13

**Authors:** Fernanda O. Novais, Augusto M. Carvalho, Megan L. Clark, Lucas P. Carvalho, Daniel P. Beiting, Igor E. Brodsky, Edgar M. Carvalho, Phillip Scott

**Affiliations:** 1 Department of Pathobiology, School of Veterinary Medicine, University of Pennsylvania, Philadelphia, Pennsylvania, United States of America; 2 Centro de Pesquisas Gonçalo Moniz, Fundação Oswaldo Cruz, Salvador, Bahia, Brazil; 3 Serviço de Imunologia, Complexo Hospitalar Prof. Edgard Santos, Universidade Federal da Bahia, Salvador, Bahia, Brazil; 4 Instituto Nacional de Ciências e Tecnologia-Doenças Tropicais, Salvador, Bahia, Brazil; Queensland Institute of Medical Research, AUSTRALIA

## Abstract

Deregulated CD8+ T cell cytotoxicity plays a central role in enhancing disease severity in several conditions. However, we have little understanding of the mechanisms by which immunopathology develops as a consequence of cytotoxicity. Using murine models of inflammation induced by the protozoan parasite leishmania, and data obtained from patients with cutaneous leishmaniasis, we uncovered a previously unrecognized role for NLRP3 inflammasome activation and IL-1β release as a detrimental consequence of CD8+ T cell-mediated cytotoxicity, ultimately resulting in chronic inflammation. Critically, pharmacological blockade of NLRP3 or IL-1β significantly ameliorated the CD8+ T cell-driven immunopathology in leishmania-infected mice. Confirming the relevance of these findings to human leishmaniasis, blockade of the NLRP3 inflammasome in skin biopsies from leishmania-infected patients prevented IL-1β release. Thus, these studies link CD8+ T cell cytotoxicity with inflammasome activation and reveal novel avenues of treatment for cutaneous leishmaniasis, as well as other of diseases where CD8+ T cell-mediated cytotoxicity induces pathology.

## Author summary

Leishmaniasis is a neglected tropical disease endemic in 98 countries and approximately 1 million new cases occur each year. Disease caused by *Leishmania braziliensis*, the main causative agent of leishmaniasis in South America, leads to skin ulcers that are difficult to heal with drugs that target the parasites. This is because disease severity seen in patients infected with *L*. *braziliensis* is largely due to the immune response that develops, rather than the number of parasites in the skin. CD8+ T cells induce cell death in the lesions of *L*. *braziliensis-*infected mice, as well as in the lesions from *L*. *braziliensis-*infected patients, which promotes disease. However, the mechanism mediating CD8+ T cell dependent pathology is unknown. Here, using studies in mice and experiments with *L*. *braziliensis* patients’ samples we show that increased disease severity is due to inflammasome activation, and furthermore that therapies that block either inflammasome activation or IL-1β ameliorate disease in mouse models of severe leishmaniasis. Based on these studies we propose a novel strategy of therapy for *L*. *braziliensis* infection and other diseases in which cytotoxicity plays a central role in promoting disease severity.

## Introduction

Granule mediated cytotoxicity is required for the clearance of several viral pathogens, as well as the killing of tumor cells [1]. However, cytotoxicity can also provoke a detrimental inflammatory response in several diseases, including experimental cerebral malaria, *Trypanosoma cruzi*-elicited cardiomyopathy and Coxsackievirus B3-induced myocarditis [2–6], and can contribute to the pathology of atherosclerotic disease, rheumatoid arthritis, chronic kidney disease, diabetes and atopic dermatitis [7–16], as well as many forms of drug-induced cutaneous hypersensitivity [17]. While we have a good understanding of the mechanism by which cytotoxicity leads to viral clearance and the control of malignant transformed host cells, how CD8+ T cell-mediated killing of infected cells leads to tissue inflammation is still poorly understood.

Cutaneous leishmaniasis, caused by an intracellular protozoan parasite transmitted by sand flies, exhibits a wide spectrum of clinical manifestations. There is no vaccine for leishmaniasis, and chemotherapeutic drugs are toxic and often ineffective. Some of the most severe forms of the disease occur in Brazil, where patients develop chronic single or multiple ulcerated lesions, and in some cases a disfiguring form of the disease called mucosal leishmaniasis. Somewhat surprisingly, these severe forms of leishmaniasis are not driven by a high parasite burden, but rather are due to an uncontrolled inflammatory response [18]. For a long time it was believed that the disease was driven by a poorly regulated CD4+ Th1 response, leading to exaggerated inflammation. However, recent findings demonstrate that the inflammation seen in *L*. *braziliensis* patients is strongly associated with granule-mediated cytotoxicity induced by CD8+ T cells [19–25], and recent studies in mice conclusively demonstrated that CD8+ T cell-mediated cytotoxicity is a cause rather than a consequence of pathology in cutaneous leishmaniasis [23] [26,27]. These findings suggest that targeting CD8+ T cell cytotoxicity for an immunotherapy might be protective, an approach far better than blocking a CD4+ Th1 response that could lead to uncontrolled parasite replication. However, to develop such a therapeutic approach requires defining the pathway that leads to severe pathology by cytolytic CD8+ T cells.

CD8+ T cell-induced apoptosis of target cells is generally not considered inflammatory, since the intracellular content of the dying cells is confined to apoptotic bodies that are rapidly engulfed by neighboring phagocytes [28]. However, there is increasing evidence that apoptosis is not always ‘silent’ and can also be immunogenic [28]. Specifically, release of “danger signals” from dying cells can activate inflammasomes, multiprotein complex sensors that regulate the processing of caspase-1 to activate pro-inflammatory cytokines such as IL-1β [29]. In support, a genome-wide transcriptional profiling of lesions from *L*. *braziliensis* patients compared to normal skin revealed that genes involved in both cytotoxicity and inflammasome activation were highly upregulated[24]. Furthermore, both inflammasome activation and IL-1β have been linked with disease severity in leishmaniasis [30], suggesting that CD8+ T cell cytotoxicity might increase inflammasome activation and IL-1β production, thereby driving disease severity.

Here we show that inflammasome activation and IL-1β release is indeed driven by CD8+ T cell-induced cytotoxicity. By employing two different murine models of infection, we found that CD8+ T cell-induced pathology depended on the NLRP3 inflammasome and IL-1β signaling, and demonstrated that the NLRP3 inflammasome is required for the high levels of IL-1β present within lesions of leishmaniasis patients. Furthermore, we demonstrated that CD8+ T cell-induced pathology could be abrogated with pharmacological inhibitors of NLRP3 or IL-1, which opens up the possibility of using several FDA-approved, commercially available drugs to ameliorate disease in patients. Together, these results provide the foundation for new strategies for treating leishmaniasis patients, as well as other diseases where CD8+ T cell-cytotoxicity drives pathology.

## Results

### IL-1β production is a consequence of actively cytolytic CD8+ T cells

**Fig 1:**
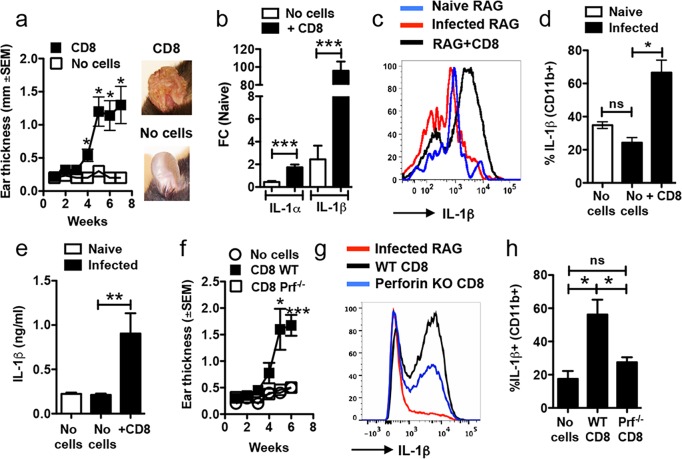
Increased IL-1β in *L*. *braziliensis* lesions is dependent on CD8 T cell cytotoxicity. RAG-/- mice were infected with *L*. *braziliensis* in the ear, and reconstituted with CD8 T cells or did not receive cells and (a) the course of infection was monitored and representative images of lesions are shown. At 7 weeks post infection mice were euthanized and (b) mRNA levels for *IL1a* and *IL1b* were assessed. mRNA data is represented as a fold change (FC) over expression in naïve mice. At 7 weeks post infection, lesions were also digested and used for flow cytometric analysis. Depicted are (c) representative histogram and (d) bar graph of intracellular staining for IL-1β. (e) Ears were cultured for 48 hours and IL-1β release was measured in the supernatants by ELISA. RAG-/- mice were infected with *L*. *braziliensis* in the ear, and reconstituted with either WT or perforin-/- CD8 T cells or did not receive cells and (f) course of infection was monitored. At 7 weeks post infection mice were euthanized, lesions were digested and used for flow cytometric analysis. Depicted are (g) representative histogram and (h) bar graph of intracellular staining for IL-1β. Representative data from one of three or more independent experiments (n = 3 to 5 mice per group) with similar results are presented. *p ≤ 0.*05* or ***p ≤ 0.*001;* ns, non-significant

In order to define the downstream mechanisms of CD8+ T cell cytotoxicity that cause immunopathology following infection with *L*. *braziliensis* we utilized our recently developed murine model [23]. As we previously reported, RAG deficient mice infected with *L*. *braziliensis* do not develop lesions in the skin despite being unable to control parasites (Fig 1A) [23]. In contrast, while RAG deficient mice reconstituted with CD8+ T cells (RAG+CD8) and infected with *L*. *braziliensis* remain unable to control the parasites [23], they now develop severe lesions over the course of weeks (Fig 1A). In addition to containing CD8+ T cells, the lesions of RAG+CD8 mice had more CD11b+ cells than control RAG mice at 7 weeks (mean number of CD11b+ cells—naïve RAG: 2.4 x 10^4^; infected RAG: 5.3 x 10^4^; RAG+CD8: 32 x 10^4^). Notably, both *Il1a* and *Il1b* RNA expression were significantly increased in RAG+CD8 mice in comparison to RAG mice, though the increase in *Il1b* was much greater than *Il1a* (Fig 1B). We next asked if pro-IL-1β protein was also expressed in the skin by flow cytometry. Infection of RAG mice with *L*. *braziliensis* did not change the expression of pro-IL-1β in the skin, suggesting that parasites alone do not induce pro-IL-1β expression at the infection site (Fig 1C and 1D). Conversely, RAG+CD8 mice had a significant increase in the frequency of CD11b+ cells expressing pro-IL-1β (Fig 1C and 1D) and there was enhanced secretion of IL-1β from ears of RAG+CD8 mice after 48 hours of in vitro culture as measured by ELISA (Fig 1E). With the exception of dendritic cells, all populations of myeloid cells analyzed, including macrophages, monocytes and neutrophils expressed more pro-IL-1β after infection in RAG+CD8 mice compared with RAG mice (S1A–S1D Fig). Notably, the pathology induced by CD8+ T cells was associated with increased recruitment of neutrophils to the skin, and the frequency of Ly6G+ cells was significantly higher in RAG+CD8 mice compared to RAG mice (S1E and S1F Fig). Therefore, the majority of cells expressing pro-IL-1β were monocytes in RAG mice, whereas in RAG+CD8 mice, neutrophils accounted for more than 70% of the IL-1β production within the skin (S1G Fig). The increased inflammation observed in RAG+CD8 mice was associated with a significant increase in mRNA levels for CCL3 and CXCL1 (S2 Fig) suggesting that CCL3 and CXCL1 might be responsible for the intense recruitment of neutrophils induced by CD8+ T cells.

The pathology induced by CD8+ T cells is dependent on granule-mediated cytotoxicity, since *L*. *braziliensis*-infected RAG mice reconstituted with perforin deficient CD8+ T cells (RAG+PRF-/-CD8) do not develop pathology (Fig 1F) [23]. Therefore, we next asked if pro-IL-1β was decreased in cells from infected RAG+PRF-/-CD8 mice. In fact, pro-IL-1β expression after *L*. *braziliensis* infection in RAG+PRF-/-CD8, though slightly higher than RAG mice infected without T cells, was significantly lower than in RAG+WT CD8 (Fig 1G and 1H), suggesting that the increased expression of pro-IL-1β in the skin is dependent on the cytolytic activity of CD8+ T cells. To determine if cytotoxicity is also necessary for pro-IL-1β expression in other skin models of inflammation, we used an imiquimod treatment model that mimics certain aspects of psoriatic lesions in which both cytotoxicity and IL-1 have been implicated in disease [31,32]. We found that imiquimod treatment (S3 Fig) increased the frequency of pro-IL-1β-producing CD11b+ cells in WT mice but not in perforin deficient mice (S3B Fig). Together, these results demonstrate that the presence of cytolytic CD8+ T cells promotes IL-1β production, which is associated with enhanced recruitment of neutrophils and other cells to the skin, many of which also express pro-IL-1β.

### Immunopathology induced by CD8+ T cells is dependent on IL-1β

**Fig 2:**
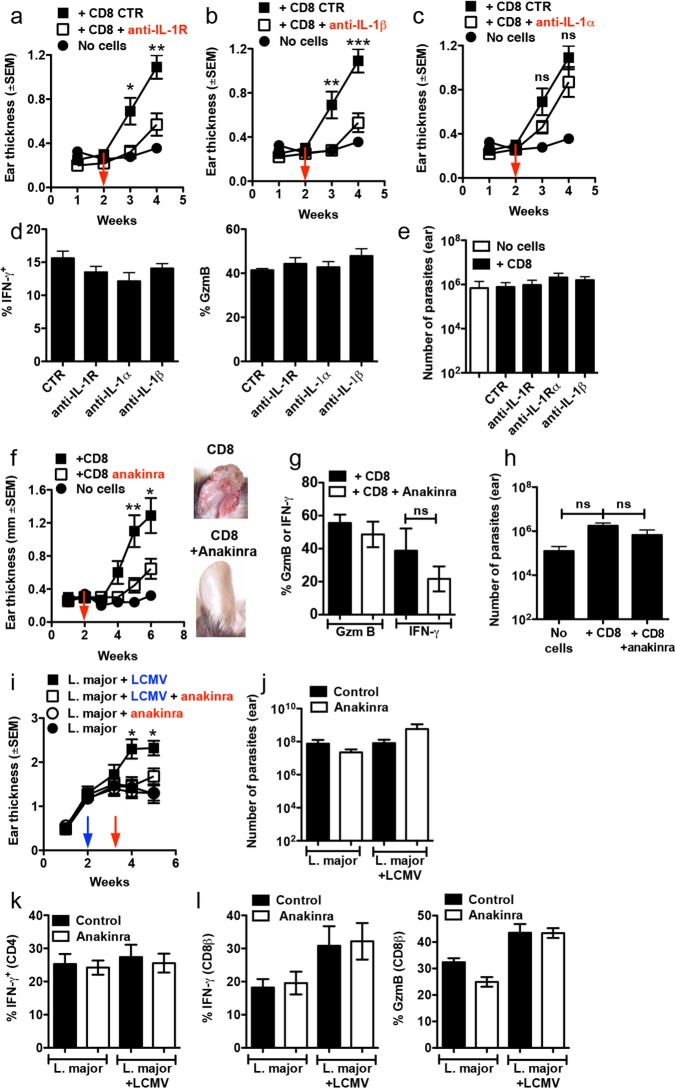
Immunopathology caused by CD8 T cells is IL-1β-dependent. RAG-/- mice were infected with *L*. *braziliensis* in the ear, and reconstituted with CD8 T cells or did not receive cells and at 2 weeks post infection mice were treated with (a) anti-IL-1R mAb, (b) anti-IL-1β mAb (c) anti-IL-1α mAb or (f) anakinra; ear thickness was assessed weekly. (d) 4 weeks or (g) 6 weeks post infection mice were euthanized and lesions were digested and used for flow cytometric analysis of intracellular IFN-γ and GzmB on CD8 T cells. (e and h) Parasite burden in the lesions. Graphs are data combined from 2 independent experiments (n = 3 to 5 mice per group in each experiment). C57BL/6 mice were infected with *L*. *major* in the ear, and 2 weeks later mice were co-infected with 2×10^5^ PFU of LCMV Armstrong strain by i.p. injection. Ten days post LCMV infection mice were treated with anakinra or were left untreated; (i) ear thickness was assessed weekly. Five weeks post infection with *L*. *major*, mice were euthanized and the (j) number of parasites in the skin was determined and lesions were digested and used for flow cytometric analysis of intracellular IFN-γ, and GzmB on (k) CD4 T cells or (l) CD8 T cells. Graphs are data from 2 independent experiments (n = 5 mice per group) with similar results are presented. *p ≤ 0.*05*, **p ≤ 0.*01* or ***p ≤ 0.*001;* ns, non-significant

To test whether IL-1β was responsible for increased disease severity, or was a consequence of increased inflammatory signaling, we monitored the extent and kinetics of lesion development in RAG+CD8 mice that were treated with anti-IL-1R, anti-IL-1β or anti-IL-1α monoclonal antibodies two weeks after infection. Notably, both anti-IL-1R and anti-IL-1β treated mice developed much smaller lesions in comparison to control mice (Fig 2A and 2B). Since IL-1α is highly expressed in the skin of mice and humans, it seemed likely that it might also contribute to the development of pathology. Indeed, recent studies show that IL-1α activated by commensal bacteria in the skin amplifies the inflammatory response in leishmaniasis [33]. However, anti-IL-1α had minimal impact on the development of pathology (Fig 2C). Importantly, blockade of IL-1α, IL-1β or both (anti-IL-1R) had no effect on CD8+ T cell production of GzmB or IFN-γ (Fig 2D), or on parasite numbers (Fig 2E). To determine if a well-established treatment for patients with IL-1 dependent inflammatory diseases might be an effective immunotherapy in cutaneous leishmaniasis, we treated RAG+CD8 mice with anakinra, a recombinant version of the IL-1R antagonist [34]. Critically, pharmacological blockade of IL-1 signaling prevented the severe CD8+ T cell-mediated pathology normally present in RAG+CD8 mice (Fig 2F), and again had no impact on GzmB or IFN-γ expression by CD8+ T cells (Fig 2G) or parasite numbers (Fig 2H). In addition, we also found that treatment with anti-IL-1R mAb decreased the lesions size of BALB/c mice infected with *L*. *braziliensis* without affecting parasite numbers (S4A and S4B Fig). Together, our data reveal that pathology induced by CD8+ T cells in the skin is dependent on IL-1β. Since blockade of this cytokine does not affect IFN-γ production or parasite control, our studies identify anakinra or monoclonal antibodies that specifically block IL-1β (such as canakinumab) as potential therapeutics for treatment of cutaneous leishmaniasis. Furthermore, since GzmB levels were not altered by IL-1 blockade, our results suggest that IL-1β production occurs downstream of CD8+ T cell activation.

In order to ask if blockade of IL-1 was effective after the onset of pathology, we started the treatment with anakinra after signs of inflammation, such as redness and thickening of the skin, had started to develop in RAG+CD8 mice. We found that treatment after pathology has already developed in RAG+CD8 mice results in slower lesion development, although it does not completely prevent inflammation (S4C Fig). Treatment after the onset of pathology did not affect parasite control (S4D Fig).

### Pharmacological blockade of IL-1 prevents pathology induced by bystander CD8+ T cells

The CD8+ T cell-mediated pathology seen in human leishmaniasis is not recapitulated in most murine models of leishmania infection. However, we recently found that co-infection with acute lymphocytic choriomeningitis virus (LCMV) in C57BL/6 mice leads to exacerbated skin immunopathology in *L*. *braziliensis*-infected mice, which depends on CD8+ T cells, and not NK cells or CD4 T cells [26]. In this model, *L*. *major* infected mice are infected systemically with LCMV two weeks post-infection. In spite of a co-infection, as in singly infected mice LCMV clearance occurs by day 8, and there is only a transient increase in the leishmania burden of co-infected animals [26]. However by 5 weeks post *L*. *major* infection, co-infected mice develop larger leishmanial lesions than do controls, with a significant increase in the frequency of neutrophils. This model, initially developed to demonstrate that viral co-infections can alter the magnitude of disease in leishmaniasis patients, allows us to probe the mechanisms of pathology mediated by CD8+ T cells in conventional animals with a full complement of immune cells. Therefore, we first determined if IL-1 was also required in this model of CD8+ T cell-dependent pathology, and treated co-infected and control mice with anakinra starting 24 days after *L*. *major* infection. As previously reported [35], the course of infection with *L*. *major* is not altered in the absence of IL-1 (Fig 2I). Importantly, however, treatment with anakinra 10 days post LCMV infection completely abrogated leishmanial-induced skin pathology (Fig 2I), despite similar parasite burdens, frequency of IFN-γ-producing CD4+ and CD8+ T (Fig 2G, 2K and 2I), and GzmB-expressing CD8+ T cells (Fig 2G and 2I). Altogether, these data demonstrate that IL-1 signaling plays an unexpected and key role in severe disease pathology in murine models of leishmaniasis driven by cytotoxic CD8+ T cells.

### Caspase-1 and the NLRP3 inflammasome are required for CD8+ T cell-mediated pathology

**Fig 3:**
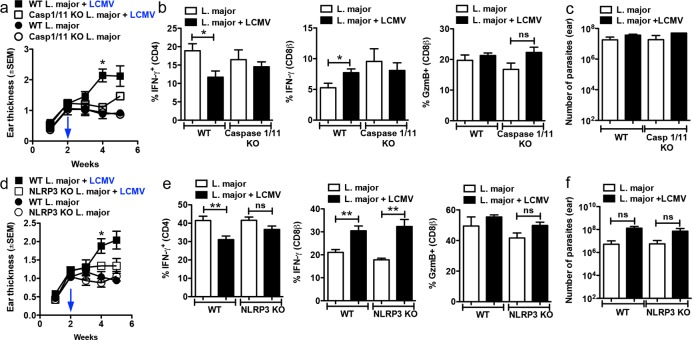
Immunopathology caused by CD8 T cells is dependent on the NLRP3 inflammasome. WT, caspase-1/11-/- or NLRP3-/- C57BL/6 mice were infected with *L*. *major* in the ear, and 2 weeks later mice were co-infected with 2×10^5^ PFU of LCMV Armstrong strain by i.p. injection; (a and d) ear thickness was assessed weekly. Five weeks post infection with *L*. *major*, mice were euthanized and the lesions were digested and used for flow cytometric analysis of intracellular IFN-γ and GzmB on (b and e) CD4 T cells or CD8 T cells. (c and f) Number of parasites in the skin was determined at 5 weeks post infection with *L*. *major*. Graphs are data from 2 to 3 independent experiments (n = 5 mice per group) with similar results. **p<0.*01*

IL-1β requires processing to become active and signal through the IL-1R [36]. There are both inflammasome dependent and independent pathways to process IL-1 and specifically, neutrophil proteases have been demonstrated to be sufficient to cleave IL-1β in situations where these cells are abundant [37] [38] [39], which is the case in *L*. *braziliensis* lesions. Therefore, to determine if the inflammasome was involved in disease caused by CD8+ T cells, we infected WT or caspase-1/11 deficient C57BL/6 mice with *L*. *major*. As previously reported [40], caspase-1/11 deficient mice infected with *L*. *major* had similar lesion development as WT mice (Fig 3A). In contrast, while WT mice developed larger lesions after co-infection with LCMV, lesions in caspase-1/11 deficient mice were smaller at 4 weeks post infection (Fig 3A). Though it is not clear if those responses are leishmania-specific, deficiency in caspases-1/11 did not affect either GzmB or IFN-γ production by CD8 or CD4 T cells (Fig 3B) and parasite numbers detected at 5 weeks post-infection remained unchanged (Fig 3C). Together, these data suggest that caspase-1/11 is required for pathology induced by CD8+ T cells and importantly does not affect parasite control. Under certain conditions caspase-11 can contribute to IL-1β processing, but regardless of the stimulus caspase-1 appears to be required [41]; at present we do not know if caspase-11 also contributes to IL-1β release in this model. Since caspase-1 plays a critical role, and can be cleaved by several different inflammasomes in order to become active [36], we next investigated which inflammasome is required for CD8+ T cell-mediated pathology.

We focused on the NLRP3 inflammasome, as *NLRP3* mRNA levels were significantly higher in lesions from *L*. *braziliensis* patients in comparison to normal skin [24], and NLRP3 can be activated by damage-associated molecular patterns (DAMPs) [42], potentially released by CD8+ T cell-induced cell death. We found that following infection of WT or NLRP3 deficient C57BL/6 mice with *L*. *major*, WT and NLRP3 deficient mice had similar lesion sizes (Fig 3D). However, while WT mice co-infected with LCMV developed severe lesions, NLRP3 deficiency decreased CD8+ T cell-mediated disease (Fig 3D). The changes in pathology were not associated with differences in the capacity of either CD4 or CD8+ T cells to make IFN-γ, or CD8+ T cells to express GzmB (Fig 3E). Importantly, the number of parasites in the skin was similar in all groups of mice (Fig 3F). These differences were not associated with a deficit in the ability of CD8+ T cells from NLRP3 deficient mice to respond since CD8+ T cells from NLRP3 deficient mice infected with LCMV alone responded as well as CD8+ T cells from wild-type mice (S5A–S5D Fig). The NLRP3 inflammasome has been previously linked with expression of inducible nitric oxide synthase (iNOS) [40], here we found no significant changes in CD11b+ cells expressing iNOS by flow cytometry (S6 Fig). Together, these results suggest that IL-1β release is dependent upon the NLRP3 inflammasome, which is particularly important as the NLRP3 inflammasome has been linked to chronic inflammation [43] and new drugs are being developed to block its activation.

### Pharmacological inhibition of the NLRP3 inflammasome prevents CD8+ T cell-mediated pathology

**Fig 4:**
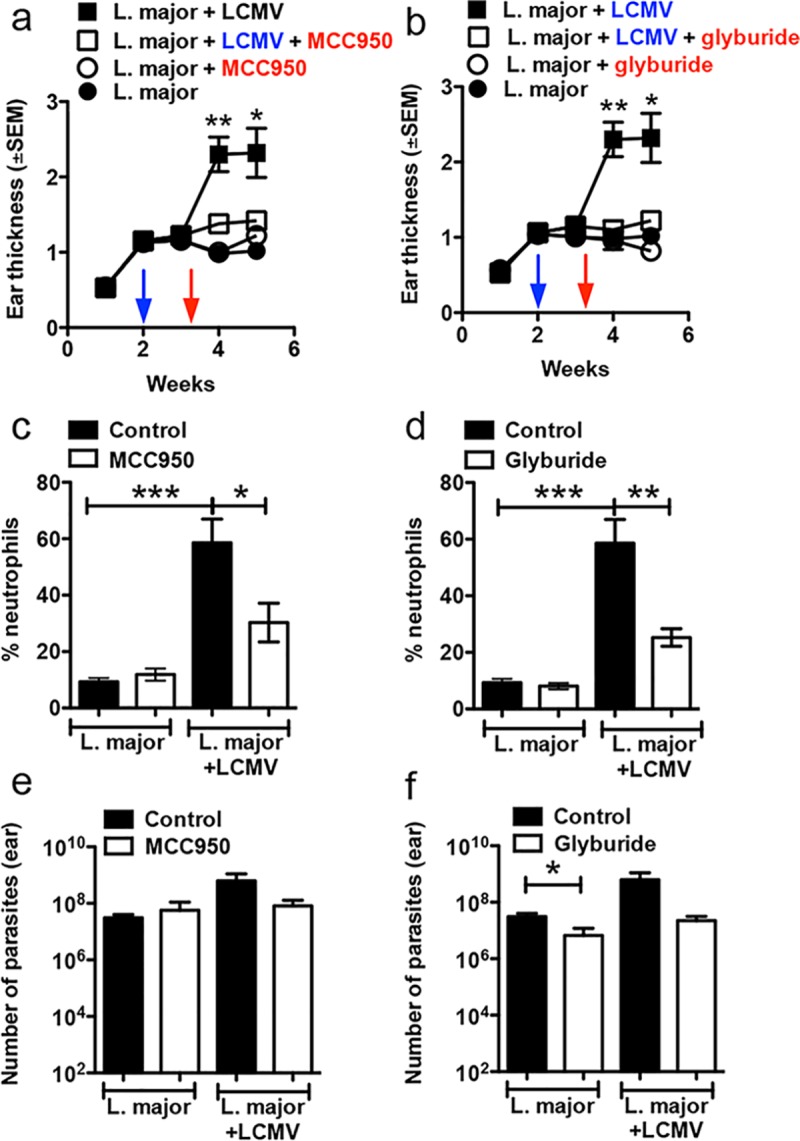
Treatment of mice with NLRP3 inhibitors dampens the immunopathology caused by CD8 T cells. WT C57BL/6 mice were infected with *L*. *major* in the ear, and 2 weeks later mice were co-infected with 2×10^5^ PFU of LCMV Armstrong strain by i.p. injection. Ten days post LCMV infection mice were treated with MCC950, glyburide or vehicle; (a and b) ear thickness was assessed weekly. Five weeks post infection with *L*. *major*, mice were euthanized and the lesions were digested and the (c and d) frequency of neutrophils in the skin was determined directly ex vivo by flow cytometry. (e and f) Number of parasites in the skin was determined at 5 weeks post infection with *L*. *major*. Results in mice are data from one experiment with 5 mice per group. *p ≤ *0*.*05*, **p<0.*01* or ***p ≤ *0*.*001*

Our results above suggest that the NLRP3 inflammasome is activated by CD8+ T cell-mediated cytotoxicity and drives disease progression. A number of small molecule inhibitors have been identified that inhibit NLRP3 inflammasome activation, for example, MCC950 and the diabetes drug glyburide [44] [45]. We therefore asked if blocking this pathway after leishmania infection could influence the progression of disease. Indeed, co-infected mice treated with MCC950 or glyburide failed to develop the severe disease seen in untreated co-infected mice (Fig 4A and 4B), while treatment with NLRP3 inhibitors had no effect on mice only infected with leishmania (Fig 4A and 4B). Critically, smaller lesions in mice treated with the NLRP3 inhibitors was associated with a decrease in neutrophils present in the skin in comparison to control mice co-infected with LCMV (Fig 4C and 4D). Inhibiting the NLRP3 inflammasome in co-infected mice had no significant effect on parasite numbers (Fig 4E and 4F), suggesting once again that reduction in disease severity was unrelated to parasite control. To confirm these results, we treated RAG+CD8 mice with glyburide or MCC950 2 weeks post *L*. *braziliensis* infection. Similar to the co-infected mice, pharmacological blockade of the NLRP3 inflammasome significantly reduced lesion development in RAG+CD8 mice (S7A Fig) without affecting parasite control in the skin (S7B Fig).

### IL-1β production is increased in lesions from leishmania-infected patients and cytotoxic effector proteins correlate with IL-1β expression

**Fig 5:**
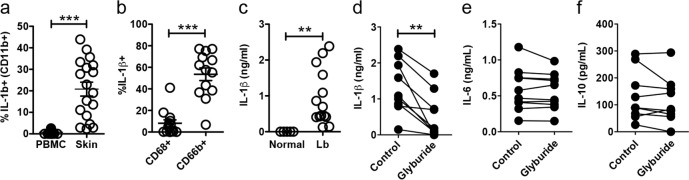
IL-1β is highly expressed in human lesions and blockade of NLRP3 prevents IL-1β release from human skin biopsies. Cells isolated from lesions or PBMC obtained from *L*. *braziliensis* patients were stained for flow cytometry directly ex vivo and depicted are (a) IL-1β+ CD11b+ cells (b) IL-1β expression within CD68+ (macrophages and monocytes) or CD66b+ (granulocytes) in the skin. Data obtained from (a) 19 PBMC or 18 skin lesions or (b) 13 skin lesions. (c) Punch biopsies from normal skin or *L*. *braziliensis* lesions were cultured for 48h and IL-1β was measured in the supernatants by ELISA. Data obtained from 5 normal skin samples and 14 lesions. *L*. *braziliensis* patients’ skin biopsies were cut in half and one half was cultured in media+DMSO and the other half with media+glyburide; 48 hours later, the presence of (d) IL-1β, (e) IL-6 and (f) IL-10 in the supernatants was determined by ELISA. Data obtained from 9 skin samples from two independent experiments. PBMC, peripheral blood mononuclear cells. **p<0.*01* or ***p<0.*001*

Using a genome-wide transcriptional profiling in *L*. *braziliensis* patients [24], we found that both *IL1A* and *IL1B* transcripts were elevated compared with normal skin (S8A and S8E Fig). Importantly, we found that levels of *IL1B* expression were positively correlated with those of *GZMB*, *GZMA* and *PRF1* (S8B, S8C and S8D Fig), whereas *IL1A* expression did not correlate with *GZMB*, *GZMA* or *PRF1*. The high levels of IL-1β mRNA correlated with increased expression of pro-IL-1β in the skin (Fig 5A). Similar to what we observed in mice, expression of pro-IL1β is different between granulocytes (CD11b+ CD66b+ cells) and macrophages/monocytes (CD11b+ CD68+ cells) in the skin of *L*. *braziliensis* patients (Fig 5B). To determine if IL-1β was processed and released by cells in leishmanial lesions, we collected lesion biopsies from *L*. *braziliensis* patients, cultured them in vitro for 48 hr, and measured the amount of IL-1β protein released into the supernatant. We found elevated levels of IL-1β in the supernatants of cultured *L*. *braziliensis* skin biopsies but not normal skin biopsies (Fig 5C). Finally, to determine if IL-1β production in human lesions was dependent on the NLRP3 inflammasome, we tested if IL-1β production by skin biopsies from *L*. *braziliensis* lesions was blocked by NLRP3 inhibition. Biopsies were again collected from patients, and then divided into two, with one half acting as a control, and the other treated with glyburide. While untreated skin lesion biopsies produced IL-1β, treatment with glyburide significantly decreased the release of IL-1β in culture (Fig 5D). Importantly, treatment with glyburide did not affect the production of other cytokines, such as IL-6 and IL-10 (Fig 5E and 5F). Together, these findings show evidence that the pathway identified in mice leading from CD8+ T cell cytotoxicity to NLRP3 inflammasome activation and subsequently IL-1β release may also be occurring in *L*. *braziliensis* patients. Thus, our results strongly suggest that targeting the inflammasome or IL-1β in humans with existing therapeutic regimens could prevent the pathology induced by excessive CD8+ T cell-mediated cytotoxicity.

## Discussion

The severity of disease progression following an infection depends not only on the pathogen burden, but also on the magnitude and type of the inflammatory response elicited. This is especially true for certain forms of cutaneous leishmaniasis, where disease progression can be relatively independent of the number of parasites and is instead driven by an exaggerated immune response. Though it is clear that cytotoxic CD8+ T cells causes immunopathology in cutaneous leishmaniasis [19] [20,21,25] [24] [23,26,27], a fundamental understanding of how cytotoxicity enhances inflammation and disease progression was undetermined. In this study, we have dissected the downstream mechanisms of CD8+ T cell cytotoxicity that exacerbates the inflammatory skin environment and found a central role for the NLRP3 inflammasome and IL-1β. Besides increasing our understanding of how cytotoxicity influences inflammation, this work provides evidence for previously unappreciated targets to suppress immunopathology in cutaneous leishmaniasis and other situations in which cytotoxicity exacerbates disease.

IL-1 has been linked to disease in many chronic inflammatory disorders and while IL-1 can have protective effects, the preponderance of data in leishmania infection indicates that excessive IL-1 is detrimental. Thus, while early production of IL-1 enhances T cell priming and promotes the development of protective Th1 responses during leishmania infection [46] [47] [48], continuous treatment of infected mice with IL-1 exacerbates disease [48] [47], and a recent study with a non-healing strain of *L*. *major* found that IL-1β was required for the chronic disease phenotype [30]. Furthermore, increased disease severity in patients infected with *L*. *mexicana* has been linked with the excess IL-1β production [49]. Taken together, the combined results of several studies indicate that while healing can occur in the absence of IL-1, the overproduction of IL-1β promotes disease severity.

The canonical pathway for maturation and release of IL-1β involves processing of pro-IL-1β by caspase-1, following activation of caspase-1 by the inflammasome. Since the NLRP3 inflammasome can be activated by DAMPs released from dying cells, and the *NLRP3* gene was upregulated in lesions from *L*. *braziliensis* patients, [24] we hypothesized that CD8-T cell mediated cytolysis drives disease progression by increasing NLRP3 inflammasome-driven caspase-1 activation. Indeed, our results show that pathology was reduced in caspase-1/11 and NLRP3 knockout mice. These results are consistent with two previous studies showing that NLRP3 inhibits protective immune responses in leishmaniasis [30,50]. In contrast, one study has reported that NLRP3 deficient mice infected with *L*. *amazonensis* are more susceptible to infection, although *L*. *amazonensis* infected mice fail to heal in the presence or absence of the NLRP3 inflammasome [40]. Since mice infected with *L*. *amazonensis* develop a poor CD4+ Th1 response [51], it may be that under these circumstances NLRP3-dependent IL-1β promotes better protection by activating the iNOS pathway of parasite control [40]. Here we found no evidence that iNOS expression is different between NLRP3 sufficient and deficient mice, suggesting that at least in CD8+ T cell-induced pathogenesis NLRP3 deficiency does limit iNOS expression in the skin.

In addition to finding that NLRP3 knockout mice developed less severe disease, we also found that inhibitors of the NLRP3 inflammasome protected mice from developing the larger lesions caused by cytotoxic CD8+ T cells. MCC950 is a small molecule that selectively inhibits the NLRP3 inflammasome and attenuates disease severity in a number of experimental autoinflammatory disease models, including murine experimental autoimmune encephalomyelitis and cryopyrin-associated autoinflammatory syndrome [44]. Glyburide, a drug commonly used in type 2 diabetes, can also prevent NLRP3-dependent IL-1β production [45]. Intriguingly, both MCC950 and glyburide prevented the development of severe lesions in leishmania-infected mice. Importantly, both genetic and pharmacological inhibition with NLRP3 inflammasome activation blocked immunopathology while keeping immunoprotective responses intact as observed by similar production of IFN-γ by T cells and equivalent parasite numbers in the skin. Particularly important for translation of our findings in mice to patients, glyburide also inhibited production of IL-1β in infected human lesion biopsies, indicating that production of IL-1β in human leishmania lesions is also dependent on NLRP3. Together, these results demonstrate that pharmacological blockade of the NLRP3 inflammasome dampens the immunopathology caused by cytotoxic CD8+ T cells, and may be an effective strategy to ameliorate disease in *L*. *braziliensis* patients.

The role of neutrophils in cutaneous leishmaniasis is complex, and depends upon particular host-parasite combinations, as well as the stage of the infection [52]. Thus, they can promote increased protection, but in other circumstances promote increased susceptibility [53–55]^,^ [56,57]. However, in chronic leishmaniasis it appears that neutrophils are primarily associated with increased disease [23,26,27,30], which occurs in the severe leishmanial lesions that develop due to excessive CD8+ T cell cytolysis. Persistent recruitment of neutrophils, which in our mouse model is associated with increased expression of CCL3 and CXCL1, drives pathology in other types of infections as well [58], by releasing matrix metalloproteases, reactive oxygen species, and myeloperoxidase all of which are significantly increased in *L*. *braziliensis* human biopsies in comparison to normal skin [24]. Notably, neutrophil-released effector molecules amplify the response of neutrophils and increase neutrophil recruitment to sites of inflammation, thereby promoting tissue damage [59]. In addition, cytokines, pathogen-associated molecular patterns and DAMPs present at inflammatory sites prolong the life span of neutrophils, contributing to chronic inflammation [60] [61] [62]. In addition to directly promoting disease, here we found that neutrophils are a major source of IL-1β during chronic infection. Thus, the recruitment of neutrophils may lead to a feed-forward loop that amplifies and sustains the inflammation in the lesions of cutaneous leishmaniasis patients.

CD8+ T cell cytotoxicity is damaging in many conditions, including drug-induced cutaneous hypersensitivity [17], experimental cerebral malaria, *Trypanosoma cruzi*-elicited cardiomyopathy and Coxsackievirus B3-induced myocarditis [2–6], and contributes to the development of artherosclerotic disease, rheumatoid arthritis, chronic kidney disease, diabetes and atopic dermatitis [7–16]. Whether the inflammation mediated by CD8+ T cell cytotoxicity is dependent on inflammasome activation in those conditions remains to be determined, although IL-1β has been implicated in driving disease severity in some of these instances [32,63–66]. Particularly relevant to our findings, *IL1b* mRNA levels are decreased in perforin or granzyme B deficient mice compared to WT mice in a mouse model of atherosclerosis [67]. Interestingly, cytotoxicity is enhanced in psoriatic lesions of human patients [31,68] and patients with psoriasis have been successfully treated with anakinra [69,70], further suggesting that cytotoxicity may drive IL-1-induced inflammation in this condition. Taken together, these findings suggest that the mechanism of inflammation induced by cytotoxic CD8+ T cells is not unique to *L*. *braziliensis* infection and may contribute to immunopathology in several other diseases.

Therapy for cutaneous leishmaniasis can be toxic and is often ineffective. This may in part be due to the fact that the drugs target the parasites but not the immunopathologic responses mediating some of the most severe forms of the disease. Using a combination of murine models and studies in cutaneous leishmaniasis patients, we have identified the NLRP3 inflammasome and IL-1β as components of the pathologic response that occurs as a consequence of CD8+ T cell cytotoxicity in severe forms of leishmaniasis. Importantly, these immune responses do not have an effect on parasite numbers. Furthermore, patients treated early during *L*. *braziliensis* infection are surprisingly less responsive to drug treatment when compared with patients that have already developed more severe lesions [71]. These patients also already have evidence of increased IL-1β [24]. Therefore, blocking IL-1β might be a valuable strategy to treat patients at the early onset of disease. Thus, blocking IL-1β with drugs currently being used clinically for other chronic diseases, such as anakinra and canakinumab, or inhibiting NLRP3 inflammasome activation, in combination with anti-parasitic drugs would ameliorate disease severity while sparing immunoprotective responses in cutaneous leishmaniasis. In summary, this work reveals a previously unrecognized link between CD8+ T cell mediated cytotoxicity and the well-known pathway of IL-1β mediated inflammation, which has important implications for patients with severe forms of cutaneous leishmaniasis.

## Methods

### Ethics statement

This study was conducted according to the principles specified in the Declaration of Helsinki and under local ethical guidelines (Ethical Committee of the Maternidade Climerio de Oliveira, Salvador, Bahia, Brazil; and the University of Pennsylvania Institutional Review Board). This study was approved by the Ethical Committee of the Federal University of Bahia (Salvador, Bahia, Brazil)(010/10) and the University of Pennsylvania IRB (Philadelphia, Pa)(812026;823847). All patients provided written informed consent for the collection of samples and subsequent analysis. This study was carried out in strict accordance with the recommendations in the Guide for the Care and Use of Laboratory Animals of the National Institutes of Health. The protocol was approved by the Institutional Animal Care and Use Committee, University of Pennsylvania Animal Welfare Assurance Number 803457.

### Patients and lesion biopsies

All cutaneous leishmaniasis patients were seen at the health post in Corte de Pedra, Bahia, Brazil, which is a well-known area of *L*. *braziliensis* transmission. The criteria for diagnosis were a clinical picture characteristic of cutaneous leishmaniasis in conjunction with parasite isolation or a positive delayed-type hypersensitivity response to leishmania antigen, plus histological features of cutaneous leishmaniasis. Prior to therapy, biopsies were collected at the border of the lesions using a 4 mm punch before therapy. Biopsies were treated with collagenase for 90 mins at 37°C/5% CO_2_, dissociated and passed through a 50 μm Medicon filter (BD phamingen). Peripheral blood mononuclear cells (PBMC) were obtained from heparinized venous blood layered over a Ficoll-Hypaque gradient (GE Healthcare), then washed by centrifugation and resuspended in RPMI media.

### Cytokine assessment in human skin

Biopsies were either cultured for assessment of IL-1β, IL-6 and IL-10 production or used directly ex vivo for flow cytometry. Biopsies from normal skin or leishmania lesions were not processed and the entire punch biopsy was cultured for 48 hours at 37°C/5% CO_2_ in RPMI supplemented with 10% human AB serum, 2 mM glutamine, 200 U/ml penicillin, and 200 μg/ml streptomycin. Where specified, the biopsies were cut into two pieces and half of the biopsies were cultured in media containing vehicle (DMSO) or 200μM of glyburide (SIGMA). Cytokine levels in the supernatants were then measured in ELISA assays (R&D Systems) according to the manufacturer’s instructions. For flow cytometry analysis, cells were stained immediately after dissociation.

### Mice

BALB/c and C57BL/6 mice (6 weeks old) were purchased from Charles River, and RAG-/- (B6.12957-RAG1^tm1Mom^) and perforin-/- (C57BL/6-Prf1^tm1Sdz^) were purchased from The Jackson Laboratory. C57BL/6 Caspase-1/11-/- mice [72] and the NLRP3-/- mice [73] were provided by Dr. Richard Flavell (Yale University). All mice were maintained in a specific pathogen-free environment at the University of Pennsylvania Animal Care Facilities.

### Parasites and LCMV

*L*. *braziliensis* parasites (strain MHOM/BR/01/BA788) and *L*. *major* parasites (Friedlin) were grown in Schneider’s insect medium (GIBCO) supplemented with 20% heat-inactivated FBS, 2 mM glutamine, 100 U/ml penicillin, and 100 μg/ml streptomycin. Metacyclic enriched promastigotes were used for infection [74]. Mice were infected with either 10^5^ *L*. *braziliensis* or 2x10^6^ *L*. *major* in the left ear, and the course of lesion progression was monitored weekly by measuring the diameter of ear induration with digital calipers (Fisher Scientific). For LCMV infections, mice were infected with 2×10^5^ PFU of LCMV Armstrong strain by i.p. injection.

### Cell purification and adoptive transfer

Splenocytes from C57BL/6 WT or perforin-/-, mice were collected, red blood cells lysed with ACK lysing buffer (LONZA) and CD8+ T cells were purified using a magnetic bead separation kit (Miltenyi Biotec). Three million CD8+ T cells were transferred into RAG mice that were subsequently infected with *L*. *braziliensis*. Mice reconstituted with CD8+ T cells received 4 injections of 250 μg of anti-CD4 within the first 2 weeks.

### Ear preparation

Infected and uninfected ears were harvested, the dorsal and ventral layers of the ear separated, and the ears incubated in RPMI (Gibco) with 250 μg/mL of Liberase (Roche) for 90 mins at 37°C/5% CO_2_. Following incubation, the enzyme reaction was stopped using 1mL of RPMI media containing 10% FBS. Ears were dissociated using a cell strainer (40 μm, BD Pharmingen) and an aliquot of the cell suspension was used for parasite titration.

### Parasite titration

The parasite burden in the ears was quantified as described previously [75]. Briefly, the homogenate was serially diluted (1:10) in 96-well plates and incubated at 26°C. The number of viable parasites was calculated from the highest dilution at which parasites were observed after 7 days.

### Flow cytometric analysis

Cell suspensions from mice were incubated with PMA (50 ng/mL), ionomycin (500 ng/mL) and Brefeldin A (10 μg /mL) (all from SIGMA) for IFN-γ and granzyme B intracellular staining. Cells suspensions were used directly ex vivo for pro-IL-1β intracellular staining. Before surface and intracellular staining, cells were washed and stained with live/dead fixable aqua dead cell stain kit (Molecular Probes), according to manufacturer instructions. Cell suspensions from human skin or PBMC were stained with flow cytometry antibodies directly ex vivo. All flow cytometry analysis was performed using the FlowJo Software.

### IL-1β assessment in mouse skin

Naive ears and *L*. *braziliensis* infected ears from RAG mice with no cells or RAG+CD8 mice were excised and the ear sheets separated and incubated intact for 48 hours in complete RPMI. Supernatants were used for mouse IL-1β ELISA (Biolegend) according to the manufacturer’s instructions.

### Antibodies and treatments

2–3 weeks post infection of RAG mice with *L*. *braziliensis* or 10 days post LCMV infection in C57BL/6 mice infected with *L*. *major*, treatment with either monoclonal antibodies or drugs commenced. Mice treated with anti-IL-1R, anti-IL-1α or anti-IL-1β (all from BioXcell) received 500 μg of antibody i.p. twice a week until the termination of the experiment. Mice were treated with 50mg/Kg of anakinra (Sobi) i.p. every day throughout the course of the experiment or 10mg/Kg of MCC950 (Sigma) and 5μM of glyburide (Sigma) i.p. every other day until the termination of the experiment. For imiquimod (Perrigo) treatment, the mouse flank was shaved the day before the first imiquimod application. WT or perforin-/- mice were treated with 62.5mg of imiquimod (5%) in the flank and in the ear for 6 consecutive days. Mice were euthanized on the 7^th^ day and ear were used for flow cytometry directly ex vivo as described above.

### Flow cytometry antibodies

Mouse: anti-CD45.2 APC-AlexaFluor 750, anti-CD11b eF450, anti-CD11c FITC, anti-F4/80 PE-Cy7, anti-CD3 eFluor 450, anti-IL-1β pro-form and anti-IFN-γ PeCy7 (all from eBioscience). Anti-CD4 APC-Cy7 and Ly6C PerCP-Cy5.5 (BD Pharmingen), anti-CD8β PerCPCy5.5 and anti-Ly6G APC (Biolegend) and anti-granzyme B APC (Invitrogen). Human: anti-CD11b PeCy7, anti-IL-1β PE, anti-CD3 APCCy7 and anti-CD8a PeCy5.5 (all from eBioscience). Anti-granzyme B APC (Invitrogen).

### RNA isolation, quantitative real-time PCR and transcriptional profiling

The ear tissue was homogenized using a tissue homogenizer (FastPrep-24, MP Biomedical), and total RNA was extracted using the RNeasy Mini kit (QIAGEN) according to the manufacturer’s instructions. RNA was reverse transcribed using high capacity cDNA Reverse Transcription (Applied Biosystems). Real-time RT-PCR was performed on a ViiA 7 Real-Time PCR System (Applied Biosystems). Relative quantities of mRNA were determined using SYBR Green PCR Master Mix (Applied Biosystems) and by the comparative threshold cycle method, as described by the manufacturer. mRNA levels for each sample were normalized to Ribosomal protein S14 genes (RPSII) and displayed as fold induction over uninfected skin. Primers were designed using Primer Express software (version 2.0; Applied Biosystems); *Il1b*, forward, 5′-TTGACGGACCCCAAAAGAT-3′, and reverse 5′-GATGTGCTGCTGCGAGATT-3′; *Il1a*, forward 5’-TTGGTTAAATGACCTGCAAC -3’, and reverse 5’-GAGCGCTCACGAACAGTTG-3’; *Ccl3*, forward, 5′- TGCCCTTGCTGTTCTTCTCT-3′, and reverse 5′- GTGGAATCTTCCGGCTGTAG-3′, *Ccl2*, forward, 5′- GCTTCTGGGCCTGCTGTTCA-3′, and reverse 5′- AGCTCTCCAGCCTACTCATT-3′; *Ccl5*, forward, 5′- GCAGCAAGTGCTCCAATCTT-3′, and reverse 5′-CAGGGAAGCGTATACAGGGT-3′; *Ccl7*, forward, 5′- AGGATCTCTGCCACGCTTC-3′, and reverse 5′- TTGACATAGCAGCATGTGGAT-3′; *Cxcl1*, forward, 5′- GCACCCAAACCGAAGTCATA-3′, and reverse 5′- CTTGGGGACACCTTTTAGCA-3′; *Cxcl4*, forward, 5′- CCATCTCCTCTGGGATCCAT-3′, and reverse 5′- CCATTCTTCAGGGTGGCTAT-3′. For transcriptional profiling, cRNA was generated from 10 normal skin and 25 lesion biopsy samples as described previously [24]. Data is deposited on the Gene Expression Omnibus (GEO) database for public access (GSE number GSE55664).

### Statistical analysis

Data are presented as mean ± standard error or individual samples. For mouse and human experiments statistical significance was determined using the two-tailed unpaired Student’s *t*-test, with the exception of paired *t*-test that was used for human experiments in which the same patient sample was compared between different treatments. Pearson correlation coefficient was used to determine correlation between LOG2 expressions of genes obtained from human skin transcriptional profiling. All statistical analysis was calculated using Prism software (GraphPad). Differences were considered significant when *p* ≤ 0.05 (*), *p* ≤ 0.01 (**) or *p* ≤ 0.001 (***).

## Supporting information

S1 Fig Neutrophils are the major source of IL-1β in the skin of RAG+CD8 mice. RAG-/- mice were infected with *L*. *braziliensis* in the ear, and reconstituted with CD8 T cells or did not receive cells. Seven weeks post infection mice were euthanized and the infected ears were digested and used for flow cytometric analysis. Depicted are representative contour plots and bar graph for intracellular staining for IL-1β within (a) neutrophils, (b) monocytes, (c) dendritic cells and (d) macrophages. Frequency of neutrophils present in the skin of infected mice was determined directly ex vivo at 7 weeks post infection. Depicted are (e) contour plots (f) bar graph for the frequency of neutrophils. IL-1β expressing CD11b+ cells were gated and the proportion of neutrophils, monocytes, dendritic cells and macrophages was determined and is represented in a (g) pie chart. Representative data from three or more independent experiments (n = 3 to 5 mice per group) with similar results are presented. *p ≤ 0.*05* or ***p ≤ 0.*001;* ns, non-significant.
(TIF)

S2 Fig Increased CCL3 and CXCL1 in *L*. *braziliensis* lesions is dependent on CD8 T cell. RAG-/- mice were infected with *L*. *braziliensis* in the ear, and reconstituted with CD8 T cells or did not receive cells. At 7 weeks post infection mice were euthanized and mRNA levels for *CCL2*, CCL3, CCL5, CCL7, CXCL1 and *CXCL4* were assessed. mRNA data is represented as a fold change (FC) over expression in naïve mice. Data from two independent experiments (n = 6 to 9 mice per group) are presented. **p ≤ 0.*01*.
(TIF)

S3 Fig IL-1β production after imiquimod treatment is dependent on perforin. (a) WT or perforin-/- mice were shaved in the flank and imiquimod or control cream was applied to the ear and flank skin for 6 consecutive days. On the 7^th^ day, mice were euthanized and the frequency of (b) IL-1β expressing CD11b+ cells in the ear were determined by flow cytometry. Representative data from 2 independent experiments (n = 3 mice per group) with similar results are presented. *p ≤ 0.*05*.
(TIF)

S4 Fig IL-1 delays lesion progression. BALB/c mice were infected with 10^5^ *L*. *braziliensis* in the ear and treated with either anti-IL-1 receptor (anti-IL-1R) monoclonal antibody or isotype (CTR); (a) ear thickness was assessed weekly and (b) parasite titration was determined 4 weeks post infection. RAG-/- mice were infected with *L*. *braziliensis* in the ear, and reconstituted with CD8 T cells or did not receive cells. At 3 weeks post infection mice were treated anakinra or were left untreated; (c) ear thickness was assessed weekly; (d) parasite burden in the lesions at 6 weeks post infection. Graphs are data from 1 (a and b) or 2 (c and d) independent experiments (n = 5 mice per group) with similar results are presented. *p ≤ 0.*05*; **p ≤ 0.*01*.
(TIF)

S5 Fig NLRP3 deficiency does not affect LCMV specific responses in CD8 T cells. WT or NLRP3-/- mice were infected with 2×10^5^ PFU of LCMV Armstrong strain by i.p. injection. 8 days post infection, mice were euthanized, the spleens were digested and stimulated with LCMV-peptide pool for 6 hours. Intracellular IFN-γ and TNF expression was determined by flow cytometry directly ex vivo. Depicted are (a and c) representative contour plots and (b and d) bar graph for IFN-γ and TNF expression within CD8 T cells. Data are representative from two independent experiments experiment with 3–7 mice per group.
(TIF)

S6 Fig Treatment of mice with NLRP3 inhibitors does not affect iNOS expression in the skin. WT or NLRP3-/- C57BL/6 mice were infected with *L*. *major* in the ear, and 2 weeks later mice were co-infected with 2×10^5^ PFU of LCMV Armstrong strain by i.p. injection. Five weeks post infection with *L*. *major*, mice were euthanized, the lesions were digested and intracellular iNOS expression was determined by flow cytometry directly ex vivo. Depicted are (a) representative contour plots and (b) bar graph for iNOS expression within CD11b+ cells. Data are representative from two independent experiments experiment with 4–5 mice per group. **p<0.*01*.
(TIF)

S7 Fig Immunopathology caused by CD8 T cells is NLRP3-dependent. RAG-/- mice were infected with *L*. *braziliensis* in the ear, and reconstituted with CD8 T cells or did not receive cells. At 2 weeks post infection mice were treated with MCC950, glyburide or vehicle; (a) ear thickness was assessed weekly; (b) parasite burden in the lesions. Graphs are data from 2 independent experiments (n = 5 mice per group) with similar results are presented. *p ≤ 0.*05*.
(TIF)

S8 Fig Cytotoxic markers with *IL1B* mRNA levels in lesions from *L*. *braziliensis* infected patients. Log2 expression of (a) *IL1B* and (e) *IL1A* in normal skin and *L*. *braziliensis* patients’ lesions. Data obtained from 10 normal skin and 25 lesions. Log2 expression of *GZMB* and (b) *IL1B* or (f) *IL1A*, *GZMA* and (c) *IL1B* or (g) *IL1A*, and *PRF1* and (d) *IL1B* or (h) *IL1A* in *l*. *braziliensis* patients’ lesions. Data obtained from 25 skin lesions [24]. **p<0.*01*; ***p ≤ 0.*001*.
(TIF)
