# Supplementary figures and images for: CD8+ T cell cytotoxicity mediates pathology in the skin by inflammasome activation and IL-1β production
Source: PLoS Pathog. 2017 Feb 13;13(2):e1006196. doi: 10.1371/journal.ppat.1006196 (PMC5325592; doi:10.1371/journal.ppat.1006196)

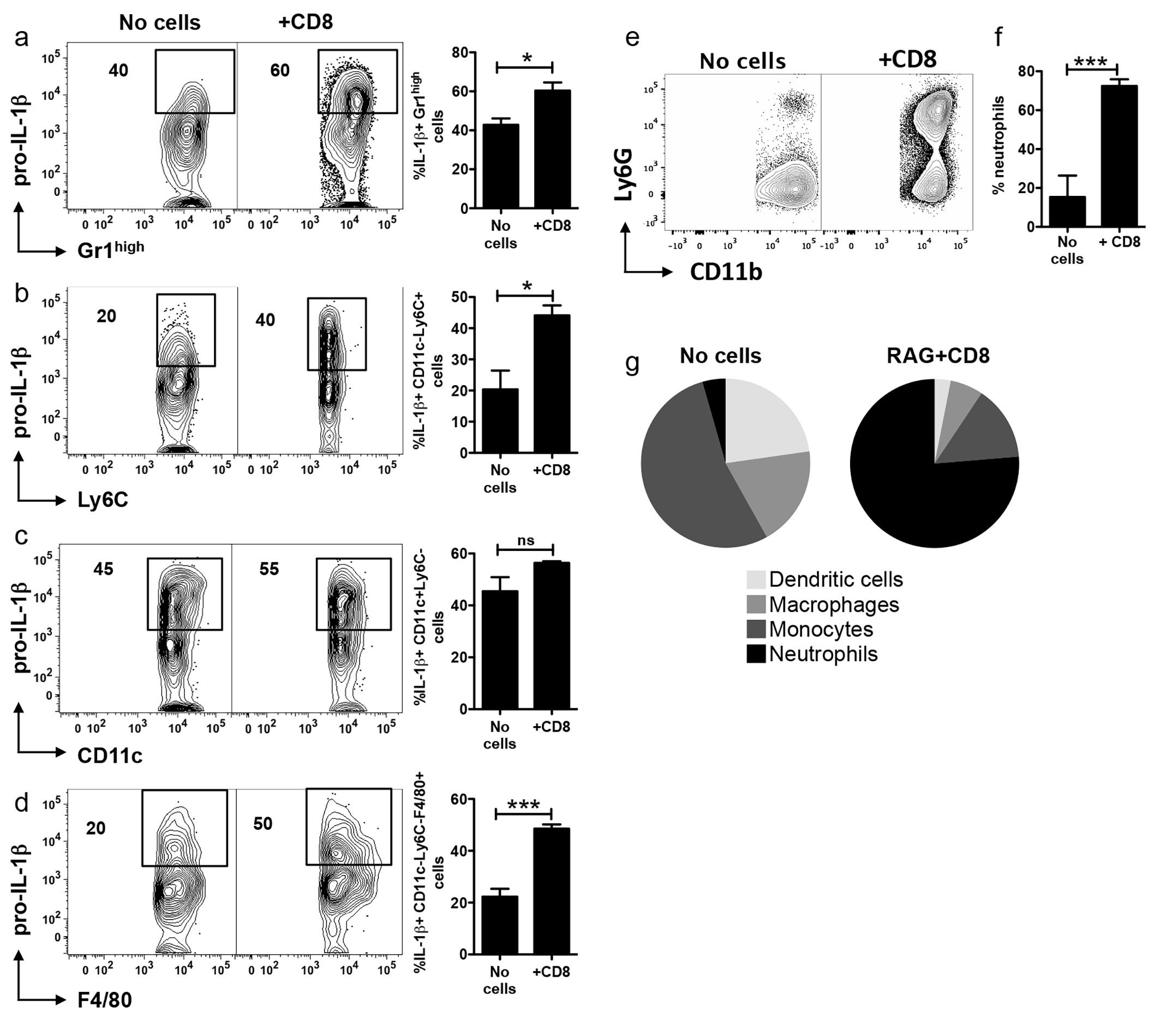

Supplement: S1 Fig — RAG-/- mice were infected with L. braziliensis in the ear, and reconstituted with CD8 T cells or did not receive cells. Seven weeks post infection mice were euthanized and the infected ears were digested and used for flow cytometric analysis. Depicted are representative contour plots and bar graph for intracellular staining for IL-1β within (a) neutrophils, (b) monocytes, (c) dendritic cells and (d) macrophages. Frequency of neutrophils present in the skin of infected mice was determined directly ex vivo at 7 weeks post infection. Depicted are (e) contour plots (f) bar graph for the frequency of neutrophils. IL-1β expressing CD11b+ cells were gated and the proportion of neutrophils, monocytes, dendritic cells and macrophages was determined and is represented in a (g) pie chart. Representative data from three or more independent experiments (n = 3 to 5 mice per group) with similar results are presented. *p ≤ 0.05 or ***p ≤ 0.001; ns, non-significant. (TIF) [file ppat.1006196.s001.tif]

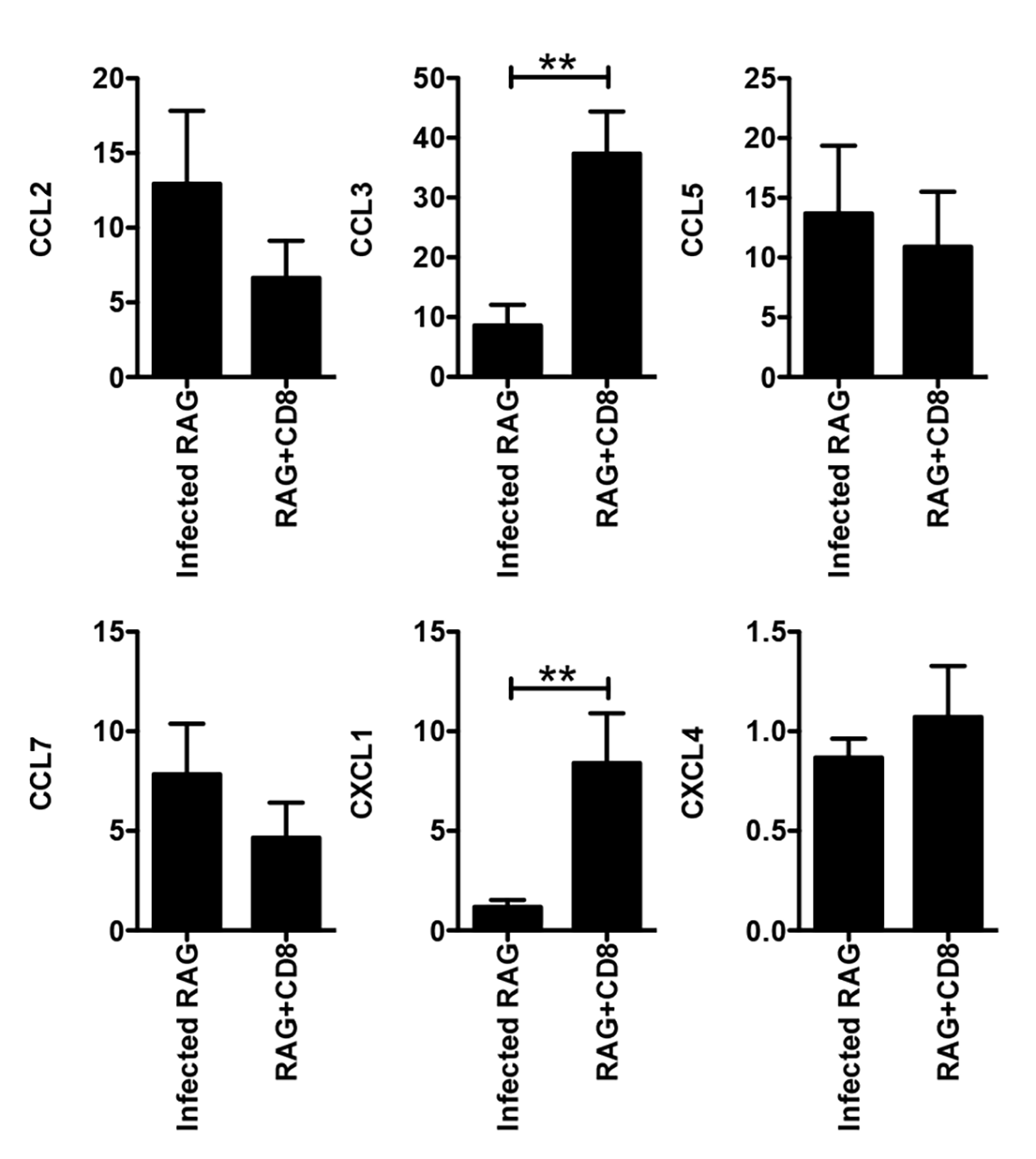

Supplement: S2 Fig — RAG-/- mice were infected with L. braziliensis in the ear, and reconstituted with CD8 T cells or did not receive cells. At 7 weeks post infection mice were euthanized and mRNA levels for CCL2, CCL3, CCL5, CCL7, CXCL1 and CXCL4 were assessed. mRNA data is represented as a fold change (FC) over expression in naïve mice. Data from two independent experiments (n = 6 to 9 mice per group) are presented. **p ≤ 0.01. (TIF) [file ppat.1006196.s002.tif]

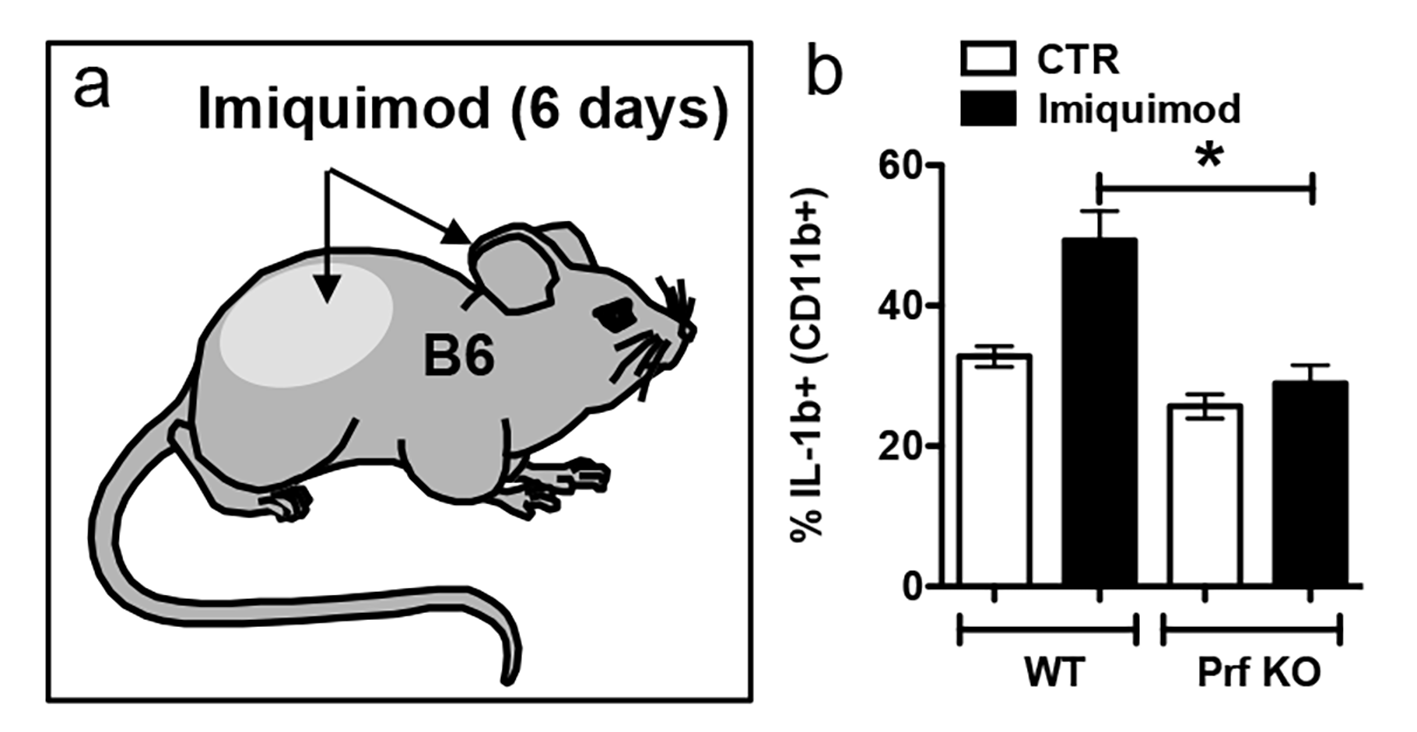

Supplement: S3 Fig — (a) WT or perforin-/- mice were shaved in the flank and imiquimod or control cream was applied to the ear and flank skin for 6 consecutive days. On the 7th day, mice were euthanized and the frequency of (b) IL-1β expressing CD11b+ cells in the ear were determined by flow cytometry. Representative data from 2 independent experiments (n = 3 mice per group) with similar results are presented. *p ≤ 0.05. (TIF) [file ppat.1006196.s003.tif]

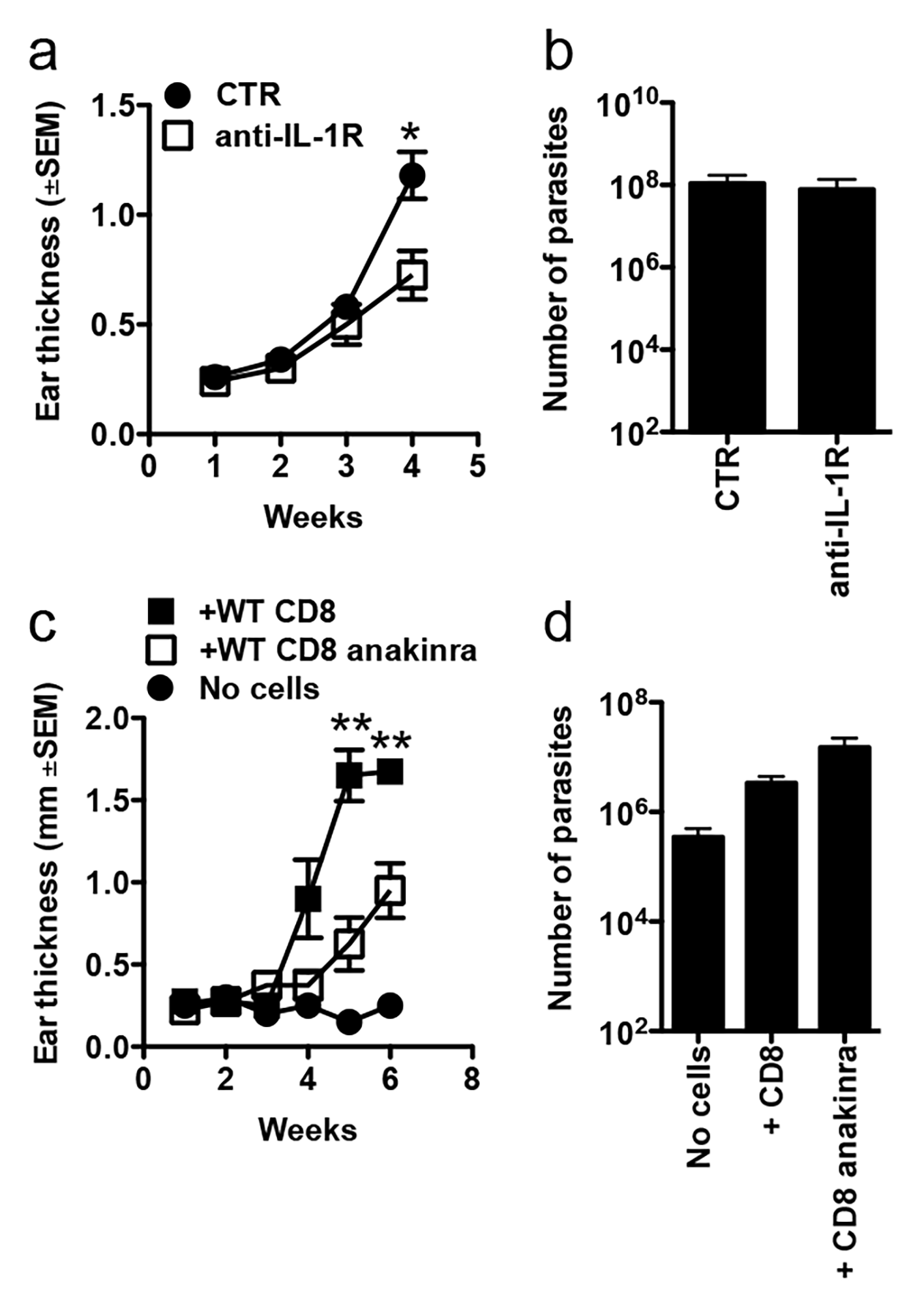

Supplement: S4 Fig — BALB/c mice were infected with 105 L. braziliensis in the ear and treated with either anti-IL-1 receptor (anti-IL-1R) monoclonal antibody or isotype (CTR); (a) ear thickness was assessed weekly and (b) parasite titration was determined 4 weeks post infection. RAG-/- mice were infected with L. braziliensis in the ear, and reconstituted with CD8 T cells or did not receive cells. At 3 weeks post infection mice were treated anakinra or were left untreated; (c) ear thickness was assessed weekly; (d) parasite burden in the lesions at 6 weeks post infection. Graphs are data from 1 (a and b) or 2 (c and d) independent experiments (n = 5 mice per group) with similar results are presented. *p ≤ 0.05; **p ≤ 0.01. (TIF) [file ppat.1006196.s004.tif]

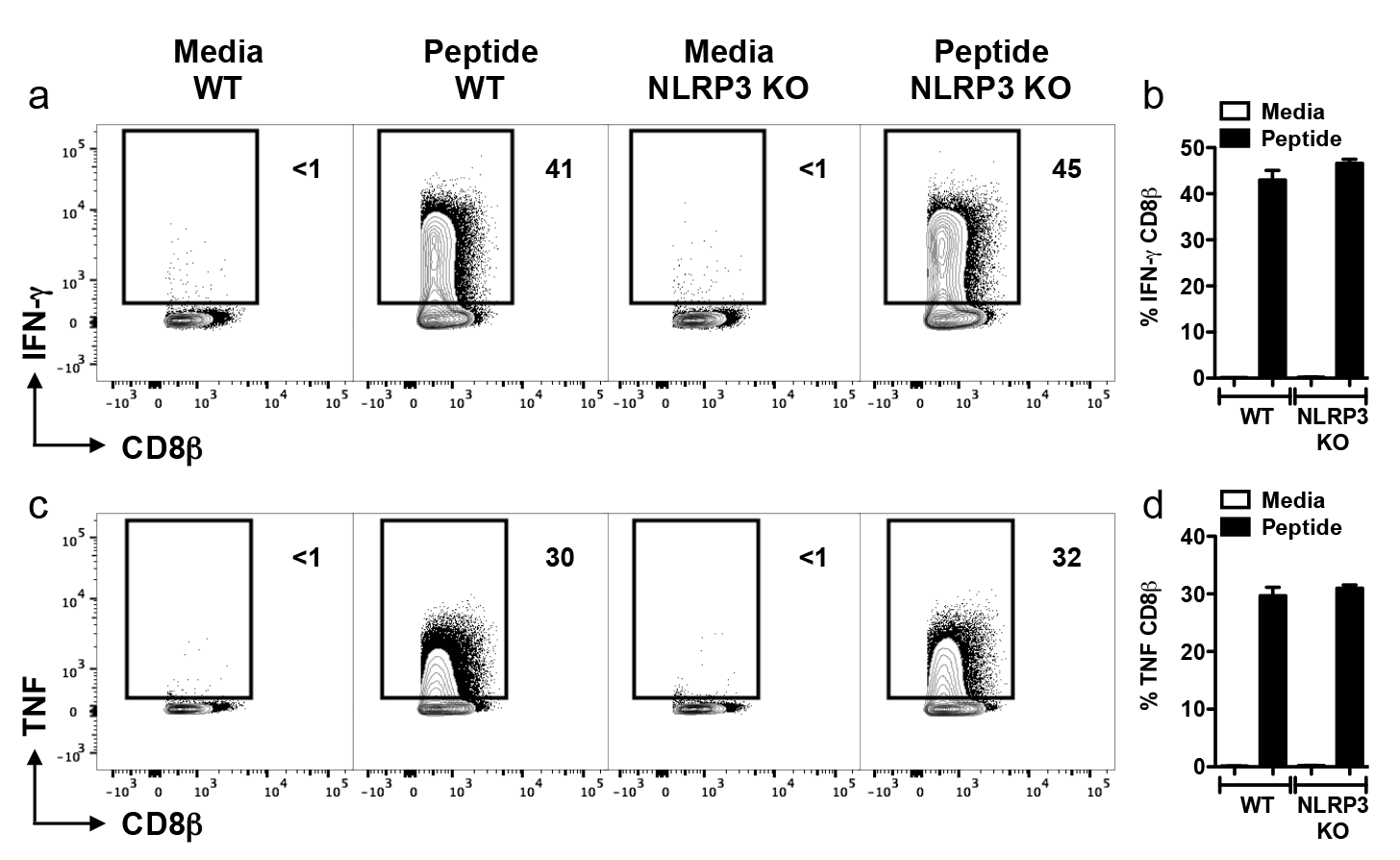

Supplement: S5 Fig — WT or NLRP3-/- mice were infected with 2×10^5^ PFU of LCMV Armstrong strain by i.p. injection. 8 days post infection, mice were euthanized, the spleens were digested and stimulated with LCMV-peptide pool for 6 hours. Intracellular IFN-γ and TNF expression was determined by flow cytometry directly ex vivo. Depicted are (a and c) representative contour plots and (b and d) bar graph for IFN-γ and TNF expression within CD8 T cells. Data are representative from two independent experiments experiment with 3–7 mice per group. (TIF) [file ppat.1006196.s005.tif]

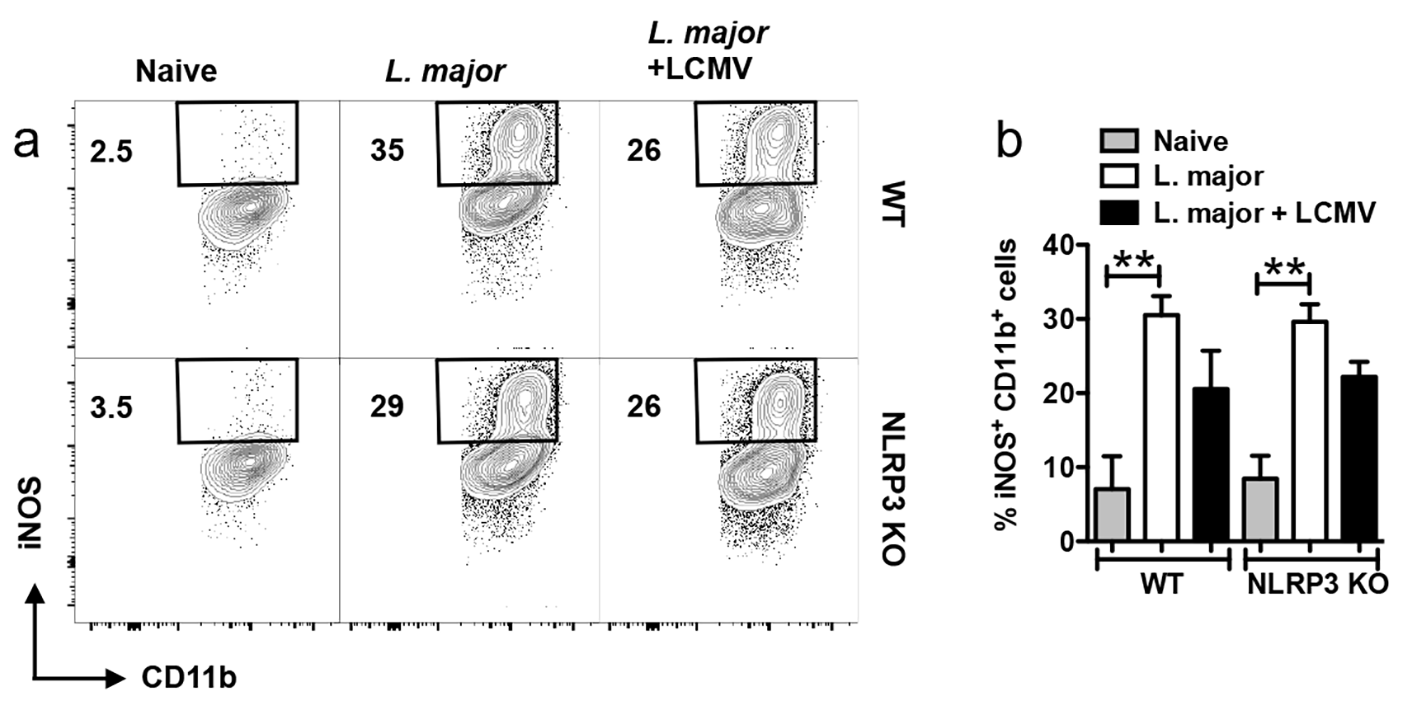

Supplement: S6 Fig — WT or NLRP3-/- C57BL/6 mice were infected with L. major in the ear, and 2 weeks later mice were co-infected with 2×10^5^ PFU of LCMV Armstrong strain by i.p. injection. Five weeks post infection with L. major, mice were euthanized, the lesions were digested and intracellular iNOS expression was determined by flow cytometry directly ex vivo. Depicted are (a) representative contour plots and (b) bar graph for iNOS expression within CD11b+ cells. Data are representative from two independent experiments experiment with 4–5 mice per group. **p<0.01. (TIF) [file ppat.1006196.s006.tif]

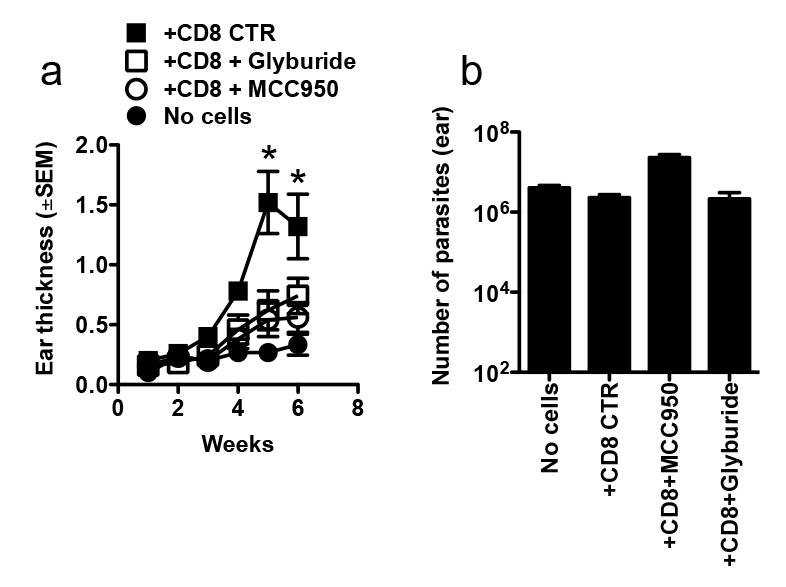

Supplement: S7 Fig — RAG-/- mice were infected with L. braziliensis in the ear, and reconstituted with CD8 T cells or did not receive cells. At 2 weeks post infection mice were treated with MCC950, glyburide or vehicle; (a) ear thickness was assessed weekly; (b) parasite burden in the lesions. Graphs are data from 2 independent experiments (n = 5 mice per group) with similar results are presented. *p ≤ 0.05. (TIF) [file ppat.1006196.s007.tif]

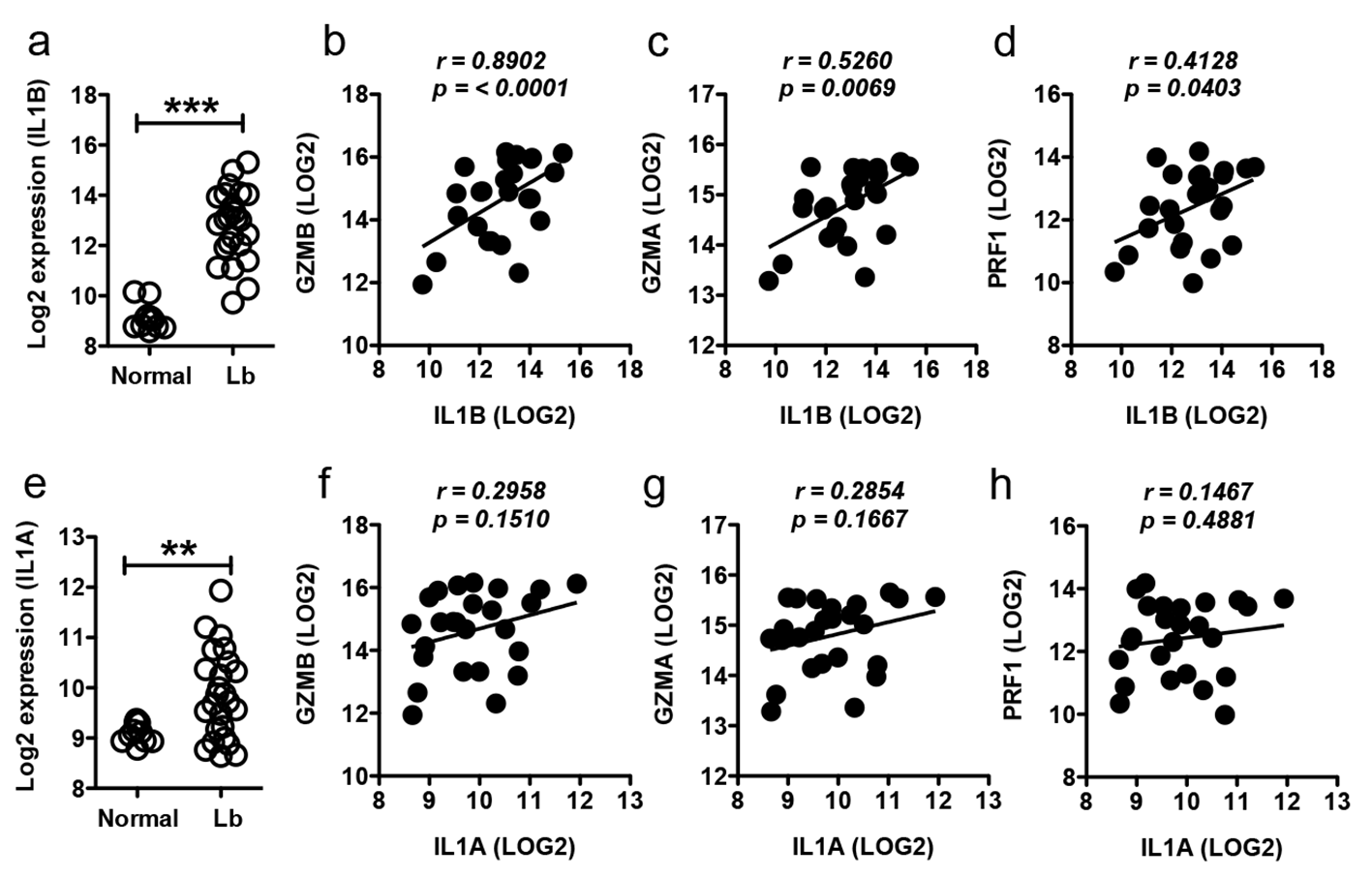

Supplement: S8 Fig — Log2 expression of (a) IL1B and (e) IL1A in normal skin and L. braziliensis patients’ lesions. Data obtained from 10 normal skin and 25 lesions. Log2 expression of GZMB and (b) IL1B or (f) IL1A, GZMA and (c) IL1B or (g) IL1A, and PRF1 and (d) IL1B or (h) IL1A in l. braziliensis patients’ lesions. Data obtained from 25 skin lesions [24]. **p<0.01; ***p ≤ 0.001. (TIF) [file ppat.1006196.s008.tif]
